# Tails of the Travelling Gaussian model and the relative age effect: Tales of age discrimination and wasted talent

**DOI:** 10.1371/journal.pone.0176206

**Published:** 2017-04-20

**Authors:** John R. Doyle, Paul A. Bottomley, Rob Angell

**Affiliations:** Cardiff Business School, Cardiff University, Cardiff, United Kingdom; Hunter College, UNITED STATES

## Abstract

The Relative Age Effect (RAE) documents the inherent disadvantages of being younger rather than older in an age-banded cohort, typically a school- or competition-year, to the detriment of career-progression, earnings and wellbeing into adulthood. We develop the Tails of the Travelling Gaussian (TTG) to model the mechanisms behind RAE. TTG has notable advantages over existing approaches, which have been largely descriptive, potentially confounded, and non-comparable across contexts. In Study 1, using data from the UK’s Millennium Cohort Study, we investigate the different levels of RAE bias across school-level academic subjects and “personality” traits. Study 2 concerns biased admissions to elite English Premier League soccer academies, and shows the model can still be used with minimal data. We also develop two practical metrics: the discrimination index (I_D_), to quantify the disadvantages facing cohort-younger children; and the wastage metric (W), to quantify the loss through untapped potential. TTG is sufficiently well-specified to simulate the consequences of I_D_ and W for policy change.

## Introduction

Older children enjoy many advantages over younger children in an age-banded cohort, typically a school year, or competition year for sports. Being older, they tend to read more fluently and have larger vocabularies, be taller, stronger, faster, more socially and emotionally aware, and so on. Such children will more likely be top of the class, selected for sports teams, and hold leadership positions, such as club presidents [1]. Each small success—actually due to chronological age, but misattributed to innate ability—will reinforce their self-esteem. It also seems obvious that any age-related difference in abilities that exist between children who are 5 and 6 years old will be greater than between those who are 10 and 11 years old. In each case, the absolute difference is one year, but in relative terms the older child has had 20%, not 10%, more time to practice, develop and mature. Following this diminishing trend through adolescence, we might expect that cohort-related differences attenuate over time until they are unnoticeable. After all, don’t late-borns attain the same average height in adulthood as early-borns? Interestingly, according to Relative Age Effect (RAE) research, the name given to this area of study, many aspects of children’s lives never auto-correct, even in the long run, leading to anomalies in career progression, earnings and well-being [2–4].

In its consequences, RAE is an unintended form of age discrimination and talent wastage. It is of concern beyond children, teachers, coaches and parents, reaching up to the managers of teaching institutions, who have the power to affect the course that RAE takes; and to policy makers, who create the wider environments in which RAE-induced wastage and discrimination may flourish, or not. The model we develop in this paper supplies the tools to better understand RAE, make better predictions and decisions. In this way, we hope the model lives up to Lewin’s [5] dictum that, "there is nothing more practical than a good theory" (p. 169).

Despite the ubiquity of RAE research, (Wikipedia lists over 100 papers), these within-cohort age-related differences have never really been modelled analytically. Past studies typically present tables of monthly or quarterly counts or percentages, perhaps with a χ^2^ test to show that the observed pattern differs from expectations with no RAE. Yet, it is difficult to compare the severity of RAE even when two sets of counts are measured in the same units, for instance months, rather than quarters or biannually. Other studies have translated birthdays into a measure of how far through the cohort-year, scaled [0,1], each child was born, with birthtime t_B_ = 0 and t_B_ = 1 being, respectively, the earliest and latest possible a child could have been born, and hence the oldest and youngest they could be within the cohort year. The individual t_B_s can then be averaged into a summary statistic ${\bar{t}}_{B}$, also known as the Index of Birthdays [6], with lower values indicating distributions positively skewed towards earlier births, and thus more severe RAE. While a single number does facilitate comparison, we show that the apparently self-evident statistic ${\bar{t}}_{B}$ is influenced by (i) the rate of talent, ability or attribute advancement, but also by (ii) the severity of selection, for instance whether the top 1% or top 0.1% are “make the grade”. In other words, past RAE research has been highly descriptive, potentially confounded, and without a method of comparison across contexts. As such, RAE research threatens to remain a series of isolated findings that cannot be accumulated in any synergistic way.

The Tails of the Travelling Gaussian (TTG) is a mathematical model of RAE that redresses the above criticisms by enabling a comparison of (i), above, uncontaminated by (ii). It is based on Gaussian distributions for a talent, ability or attribute, which collectively we refer to as *qualities*. There is a Gaussian for the youngest children in the cohort, a Gaussian for the oldest children, and one for every birthday in between. Comparing the upper tails of such Gaussians allows us to estimate the probability that children born on different days will exceed a particular criterion (e.g., selected into a soccer academy, or rated by teachers “well above average”). We can also work backwards from known selection probabilities at different birthtimes of the year to infer the unobserved rate at which a quality develops within the cohort, *i*.*e*., how fast the Gaussian travels. In practice, this latter approach is likely to be most popular, given the format of available empirical data.

With knowledge of the relative rates of advancement, the TTG enables comparison of widely different qualities which could not otherwise be compared. In addition, the *form* of advancement can be traced. Does it develop at a constant rate, or are there critical periods for learning, akin to growth-spurts and puberty? We can also derive related statistics which will illuminate issues such as the degree of age discrimination operating, or the amount of talent being wasted in a particular situation. Both should interest policy makers wishing to mitigate the negative consequences of RAE. Also, because the TTG is an analytic model, it can be used to simulate changes in policy, such as moving to half-year cohorts, relaxing selection criteria, or delaying entry into talent schools, as well as being able to measure critical outcomes of policy changes.

Next, we briefly review the literature, present the theoretical underpinnings of the TTG model and elaborate on its advantages over ${\bar{t}}_{B}$, mean birthtime. We then illustrate how the TTG model adds value and insight using real data from different domains (sports, education and personality). This also shows how the model can be employed in practice, particularly when the data is compiled in different formats (frequency tables and selection probabilities). Finally, we discuss implications for theory and practice, along with directions for future research.

## Tails of the Travelling Gaussian (TTG)

### Background literature

The literature charts a consistent set of RAE effects from Kindergarten [7], through early school [8], middle school [9, 10], to upper school [11] and university [12]. Children born at the start of the academic year do better academically, whereas those born at the end of the year do less well and are more likely to be diagnosed with attention deficit / hyperactivity disorder (ADHD), be referred with special needs, or even present with psychiatric problems [13].

The intrinsic age differences in RAE, so evident at an early age, tend to attenuate as the age-cohort grows up. But a diverse and consistent body of work suggests that such RAEs do not disappear altogether [2]. Studies have reported lower self-esteem among those born later in the academic year [14]; higher rates of suicide and obesity [15, 16]; lower qualities of leadership [1]; and lower educational attainment [17, 18].

One of the intrigues of RAE is seeing these patterns persist into adulthood, long after they should have disappeared. In terms of elite performance in adult life, more early-borns attend UK’s premier Oxford and Cambridge universities. The US Congress is also overrepresented by those born earlier in the cohort [4], as are corporate CEOs [19], and UK Nobel laureates in science [20]. RAE exists among NHL professional ice hockey players [21], in Serie A, Italy’s top soccer league [3], and Olympic athletes [22].

But, the clear message of RAE overrepresentation through into adulthood does not extend quite so straightforwardly to earnings. Larsen and Solli [23] studied Norwegians born in the 1940s and showed that earnings were greater for early-borns, but only until their mid-40s, after which time late-borns tended to earn more. Similarly, findings about wages among elite performers have been equivocal; Bryson, Gomez and Zhang [21] showing that later-borns tend to earn more (ice hockey); whereas Rossi and Fumarco [3] that later-borns earn less (soccer). What does seem unequivocal is that early borns have a more advantaged path into adulthood, whether they fully capitalise on it or not.

One mechanism behind the persistence of RAE are the "self-fulfilling prophesies" described in Hancock, Adler and Côté [24]. Doors may be opened or closed on the basis of RAE. The teacher may give the older children leading roles in the school play, select them for sports teams, and assign them to more able work groups. Each creates differences in the exposure necessary for growth. Parents may reinforce this through "skewed enrolment bias", by failing to engage their cohort-younger children into extra-curricular activities. These “self-fulfilling prophesies” may be internalised too, so "once expectations are placed upon an individual, that individual typically acts congruently with those expectations" [24], Thus, teachers, parents, peers, and children themselves may all be working to reinforce feedback loops, which perpetuate RAE and undermine or enhance positive self-esteem.

Because children attend school for most of their childhood, no matter how behind they seem at first, there is always a chance that eventually they can catch up, revise their public- and self-image, and thereby realise more of their true potential. Age should thus attenuate the severity of RAE found in early childhood. In contrast, “talent” schools aim to accelerate the latent ability of their pupils, who are often selected at an early age when intrinsic RAE is greatest. Moreover, if the school is successful in its *raison d'être*, it will introduce a gulf in performance between those inside and those outside the institution. Thus, outsiders will find it increasingly difficult to catch up and gain admission as time passes and the gulf widens. The age profile of talent schools’ pupils thus tends to be frozen in the state it was at first entry. Soccer academies are one such talent school that we will explore in study 2.

Giving evidence to these claims, among 7 and 8 year olds, a year will make a big difference to the talent a scout perceives a boy to have; thus at the U9 (under-9) level, soccer academies are typically top-heavy with boys born at the start of the competition year. But once established, we claim this “bias” will be largely impervious to outside influence. Accordingly, we should find RAE bias at the highest levels of youth soccer, which is not so very different from that found lower down the ages; and this perseverance of pattern *is* typically found. For instance, Del Campo et al. [25], in their Figure 6, found in Spanish soccer, that the pattern of RAE bias observed at U13, U15, U18 levels was almost identical to the pattern at U11 level. Deprez et al. [26], in their Table 1 found similar levels of RAE bias for a Flemish (Belgian) sample at U10-U11, U12-U13, U14-U15, U16-U17, and U18-U19, namely the ratios of players born in Quarter 1 to Quarter 4 were 2.9, 3.2, 3.1, 2.4, and 2.7, respectively. Finally, Lovell et al. [27], in their Table 1 found no diminution of RAE between U9 and U19 for boys in lower league, English soccer development programmes. Thus, the age profile of the entire academy is likely to look much like the age profile at 7–8 years, as indeed it should, because that is where it originated, remaining largely undiluted with time.

**Table 1:** Children’s speaking and listening ratings disaggregated by month-of-birth.

	Well Below	Below	Average	Above	Well Above	Cumulative Frequencies				
	1	2	3	4	5	≥ 1	≥ 2	≥ 3	≥ 4	≥ 5
Sep	3	39	161	195	72	470	467	428	267	72
Oct	13	34	169	171	61	448	435	401	232	61
Nov	6	36	197	168	66	473	467	431	234	66
Dec	9	52	206	158	44	469	460	408	202	44
Jan	9	60	168	168	50	455	446	386	218	50
Feb	13	53	183	142	36	427	414	361	178	36
Mar	11	52	195	140	40	438	427	375	180	40
Apr	12	49	186	140	31	418	406	357	171	31
May	17	59	209	121	30	436	419	360	151	30
Jun	22	60	213	115	25	435	413	353	140	25
Jul	22	71	171	105	33	402	380	309	138	33
Aug	13	64	211	79	24	391	378	314	103	24

In contrast to sports, education is primarily cognitive rather than physical, is not overtly competitive, and is explicitly measured, for instance by public examination. Education data usually covers a representative sample of the population, rather than a highly selected upper tail of extreme-ability children, as is often the case with sport. Chess occupies an interesting niche between these extremes, being both competitive *and* cognitive, with ability explicitly measured using the Elo system [28]. Breznik and Law’s [28] analysis of expert chess players revealed the usual RAE downward trend in frequency across all quarters of the year (Jan-Dec) for boys and women; but for girls and men, while the downward trend was present across quarters 1 to 3, there was a pronounced upturn in quarter 4 born players. Gobet and Chassy [29] reported a similar upturn in younger, later born chess players which they interpreted as a genuine seasonal effect.

This raises important issues for any analytical model and its associated estimation technique. If the distribution of birthdates were genuinely driven by season, we should expect no great discontinuity across the competition cut-off date, the trend being a wave that repeats year on year. But, if the data are driven by RAE, we should expect a discontinuity, the trend having the appearance of a saw-tooth function. Clearly, any estimation technique must be capable of distinguishing between these two distinct profiles, and specifically testing for both linear and non-linear relations in the distribution of birthdates. Our two real-world examples will illustrate the application of such an approach.

### Key concept: Continuum of between-to-within age variability

In each area of activity, there will be a disparate spread of qualities (talents, abilities, attributes), even among children born on the same day. The larger the difference a year makes to the development of a quality relative to the overall spread among same-birthday children, the more it will matter whether a child is the youngest or oldest in their cohort. A good example is that children get taller as they age, conferring advantage in size-critical activities. Conversely, the smaller the rate of annual advancement relative to the spread of qualities among same-birthday children, the less it will matter whether a child is the youngest or oldest in their cohort. For example, children produce more melanin as they grow up, improving their “ability” to avoid sunburn [30]. But, the effect is so small that whether a child is younger or older in their cohort makes little difference to how much sun they can withstand before burning—what matters is the inherent variability in skin tone *at* or *within* an age, which can differ hundredfold according to Rees [31].

We can thus think of a continuum with *age-sensitive* differences, such as height dominating towards one end, and *age-insensitive* differences, such as skin tone dominating at the other. This continuum captures the relative size of *between-age* advancement to *within-age* variability. At points along the continuum we should find the heterogeneous *qualities* to which RAE applies. Clearly, some qualities are more passively acquired (e.g., height, skin tone), while others require practice and effort for the latent talent to be manifest (e.g., reading, musical ability). Likewise, some qualities are more objectively measurable (e.g., running speed) and others less so (e.g., emotional maturation, soccer skills). Nevertheless, the TTG model can map all these qualities onto the age-sensitivity continuum, and capture in a single measure, namely the rate of Annual Advancement (A), how much difference a year makes. We now explain how A can be derived from our proposed mathematical model.

### Tails of the Travelling Gaussian (TTG) model

**Fig 1:**
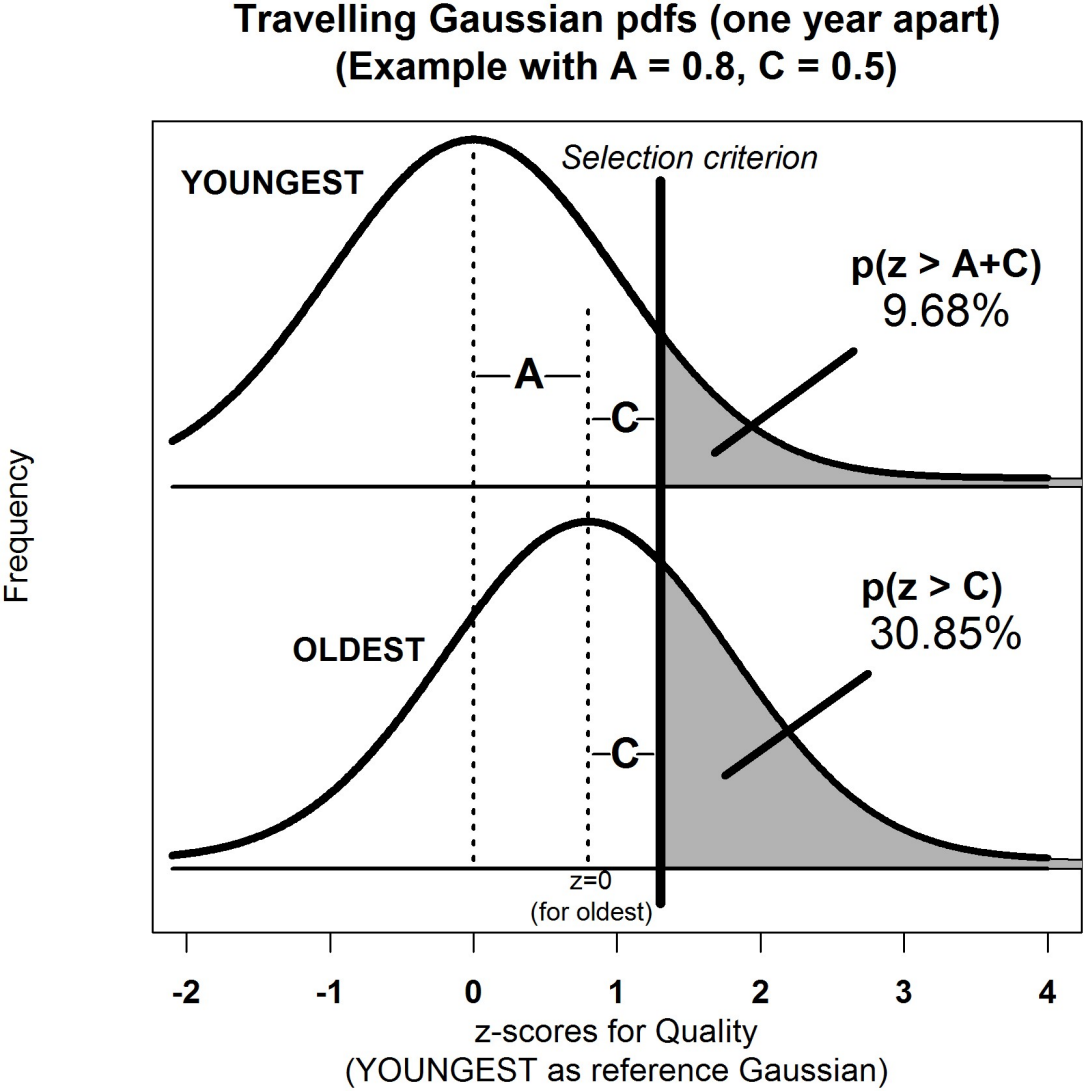
Standardized normal distributions for *quality* of oldest and youngest children in an age-banded cohort. Oldest born at t_B_ = 0, youngest born one year later at t_B_ = 1. A is the rate of annual advancement. C is the selection criterion.

Fig 1 shows the Gaussian distribution for a quality expressed in standardized units (z-scores) for the oldest children of a cohort born at time t_B_ = 0; and the youngest born one year later at time t_B_ = 1. In general, if a quality advances by A units per annum, the distributions for youngest and oldest will be ~N(0,1) and ~N(A,1) respectively. With knowledge about children at points in between, we can infer *how* the Gaussian *travels* from youngest to oldest. For instance, if the quality advances at a constant rate with time t_B_, then its distribution will be ~N(t_B_A,1) for 0 ≤ *t* ≤ 1.

Imagine that a professional soccer club wants to recruit and train youth goalkeepers, but only if they meet or exceed some minimum height requirement denoted by the vertical Selection Criterion in Fig 1. The location of this line is measured by the distance A+C, relative to the midpoint of the Gaussian distribution for the youngest children, as shown in the top half of Fig 1. Likewise, this line is measured by the distance C, relative to the midpoint of the Gaussian for the oldest children, as shown in the bottom half of Fig 1. Thus, the Gaussian will travel a distance of A units (standard deviations) to the right during the course of a year. In this case, A measures how much children grow in height, on average, per annum. Obviously, the younger you are, the more difficult it is to meet the height requirements of the professional soccer academy. Whereas for the oldest children this criterion is met by those who are only slightly taller than average, at z ≥ C, the youngest children need to be a lot taller than average for their chronological age, at z ≥ A+C.

**Fig 2:**
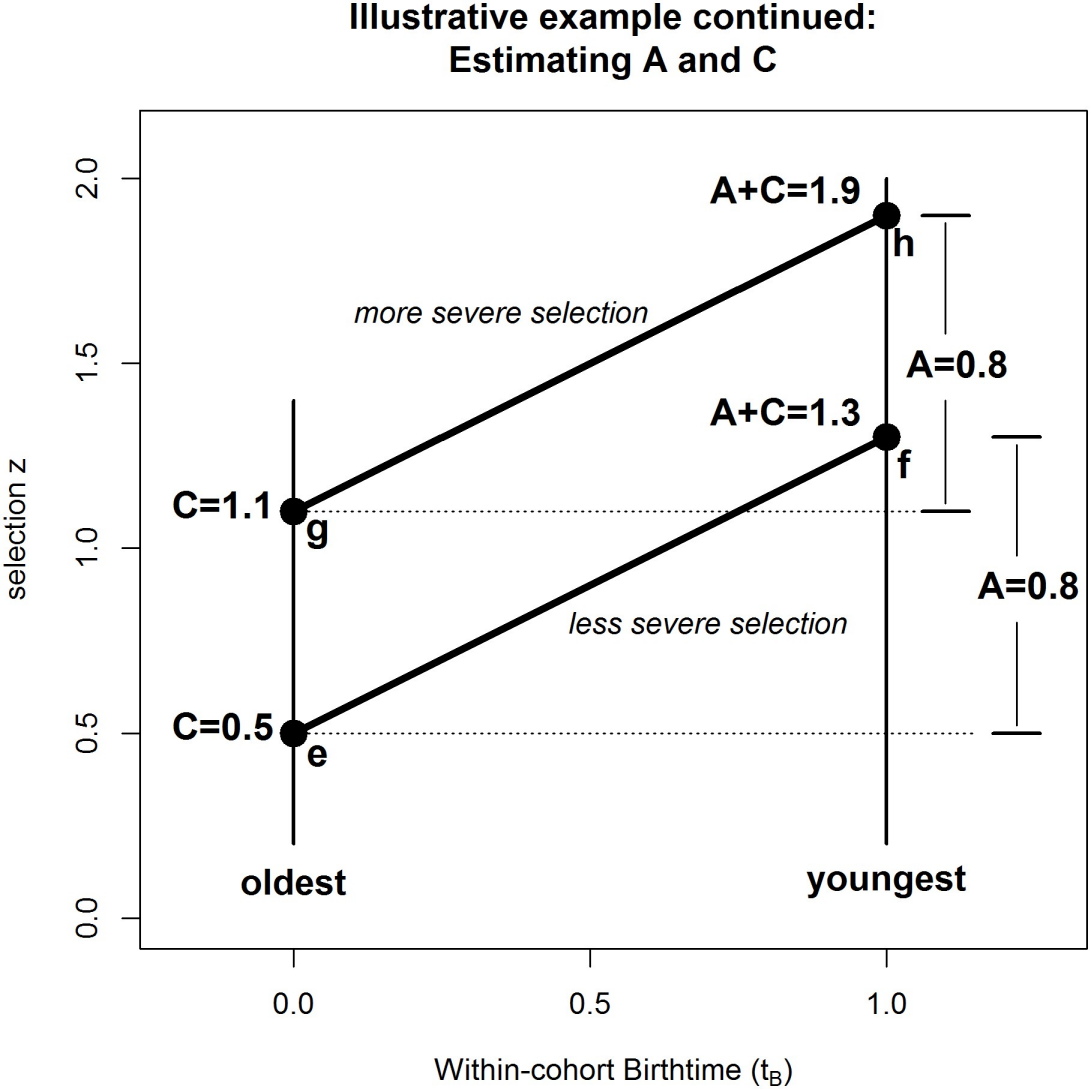
Illustrative example continued. Calculating A and C (as z-values) from probabilities of selection for oldest and youngest in a cohort.

Given empirical data on selection percentages, we can work backwards and determine what A and C actually are. Suppose, as in Fig 1, we observe 9.68% and 30.85% of the youngest and oldest children meet the height requirements, as denoted by the shaded areas. Using knowledge of the standard normal / Gaussian distribution, we can infer the effective z for the youngest children is 1.3 (A+C) because p(z ≥ 1.3) = .0968; mathematically: z = Φ^-1^(1–0.0968), where Φ is the cumulative distribution function (cdf) for the standard normal, N(0,1), that maps z-values onto p-values; and Φ^-1^ is its inverse, that maps p-values onto z-values. This corresponds to point f in Fig 2. Likewise, as 30.85% of oldest children meet the height criterion (corresponding to point e in Fig 2), we can infer their effective z is 0.5 (C) because p(z ≥ 0.5) = .3085. By subtraction, we conclude A = 0.8. Thus, height (as a quality) increases by 0.8 standard deviations on average during this cohort-year.

Now imagine that the soccer club raises their minimum height requirement. This more severe selection criterion will shift the heavy black line further to the right in Fig 1. If only 2.87% of the youngest and 13.57% of the oldest children are selected, we would back-translate these probabilities into z-scores of 1.9 (A+C) and 1.1 (C) respectively, corresponding to points h and g in Fig 2, and would again infer that A = 0.8. In the world of constructed examples, both scenarios give exactly the same value of A. For real-world empirical data they *might* not. But, one advantage of analysing different severities of selection on the same population pool is that, via a process of triangulation, we obtain multiple estimates of the rate of annual advancement (A) for the quality of interest, plus a measure of its uncertainty (standard error). The mapping between known areas (probabilities) in Fig 1 and the inferred points e, f, g, h in Fig 2 is abbreviated as follows:

Point (e) p(z ≥ 0.5) = 0.3085, thus C = 0.5 for *less* severe selection.
Point (f) p(z ≥ 1.3) = 0.0968, thus A+C = 1.3 for *less* severe selection.
Point (g) p(z ≥ 1.1) = 0.1357, thus C = 1.1 for *more* severe selection.
Point (f) p(z ≥ 1.9) = 0.0287,, thus A+C = 1.9 for *more* severe selection.

### Ambiguities in mean birthtime (${\bar{t}}_{B}$) as a measure of RAE severity

Earlier we claimed that the mean birthtime, ${\bar{t}}_{B}$, was jointly influenced by the rate of ability advancement (A) and the severity of selection (C), thereby limiting its usefulness in RAE research. We now substantiate this claim by showing that ${\bar{t}}_{B}$ decreases as: (i) selection becomes more severe (*i*.*e*., C increases, holding A fixed), and (ii) quality advances at a faster rate (*i*.*e*., A increases, holding C fixed). Returning to Fig 1, we know that when C = 0.5, (A = 0.8 = fixed), 31.85% of the oldest and 9.68% of the youngest are eligible for admission into the soccer academy. Thus, children born at the very start of the year are 3.18 (= 31.85 / 9.68) times more likely to meet the goalkeeping height requirements than children born right at the end. When the selection criterion is more severe, and only taller goalkeepers are recruited, (C = 1.1; A = 0.8 = fixed), children born at the very start of the year are now 4.72 times (13.57 / 2.87) more likely to be selected than those born right at the end. Thus, as the heavy black line denoting the selection criterion (C) shifts further to the right, the disparity (odds ratio) between oldest and youngest increases and the distribution of those selected skews more heavily in favour of the oldest, near t_B_ = 0; and so ${\bar{t}}_{B}$ decreases. This is true not just for A = 0.8, but is a general rule for any fixed A, easily verifiable via statistical tables, a spreadsheet, or formally in mathematics.

Likewise, the bigger the difference a year makes to the development and advancement of a quality, (A increases, holding C fixed), the more the two Gaussians will be pulled apart, but only to the disadvantage of the youngest children. Returning to Fig 1, notice that for the oldest children, the probability of being selected p(z ≥ C) is independent of A. However, for the youngest children, the selection probability *will* decrease as A increases p(z ≥ A + C), with their Gaussian starting further to the left, and so the disparity (odds ratio) between oldest and youngest will increase. For instance, when C = 0.5 (fixed), 31.85% of the oldest are always selected, regardless of A. If quality advances by 0.8 standard deviations annually, as given in Fig 1, then 9.68% of the youngest are selected (z = 1.3; 0.8 + 0.5). But if ability advances at a faster rate, say A = 1.2 (not shown in Fig 1), now only 4.46% (z = 1.7; 1.2 + 0.5) of the youngest will “make the grade”. Thus, children born at the very start of the year are no longer 3.18 but 6.92 (= 30.85 / 4.46) times more likely to meet the goalkeeping requirements than those born right at the end. Again, the distribution of those selected skews more heavily in favour of the oldest near t_B_ = 0; and so ${\bar{t}}_{B}$, decreases.

It follows that any given ${\bar{t}}_{B}$, being affected by both A and C, is not measuring the underlying severity of RAE alone (as captured in A); nor is it measuring the severity of selection alone (as captured in C). In fact, it is measuring an ambiguous combination of them both. For this reason, the TTG is preferred.

None of this is to deny a place for ${\bar{t}}_{B}$ in RAE research. Sometimes we might want to describe the degree of RAE existing in a situation, and not worry too much (yet) how much it is due to A or to C; or we may find it difficult to estimate the size of the population pool, and identify C and thus isolate A. In cases where we can’t or don’t want to partition ${\bar{t}}_{B}$ into its A and C components, then ${\bar{t}}_{B}$ would be an attractive, easily understood portmanteau statistic to use, though with caution. Finally, note that t_B_ itself is an objective measure of where in the year someone’s birthday lies, or where to locate the midpoints of the months or quarters of the year. The point of contention is not with t_B_, but what can be inferred from using ${\bar{t}}_{B}$.

## Example studies

We now turn our attention to two real-world empirical datasets. This serves three purposes. First, each provides a working example of how the TTG model can be practically used to measure RAE. Second, each serves to illustrate how flexible the model is in different contexts, including the type and amount of information it requires. Finally, it enables us to introduce two important practical metrics for evaluating the extent of RAE discrimination in any particular situation.

The first example uses teacher ratings of pupils from the UK’s Millennium Cohort Study (MCS, [32–24]). The analysis follows the illustrative example above for, except that children fall into one of twelve birth-months, instead of a binary category (oldest versus youngest); and children are rated “as if” at four levels of selection severity, instead of just two (C = 0.5 and 1.1). The TTG model is used to map a range of curricular and non-curricular qualities onto their respective A values, thereby facilitating comparison across disparate domains.

The second example uses data concerning English Premier League soccer academies, and is an example of RAE in talent schools. The data is scant (percentage of academy boys born in each third of the year), and the starting population pool is unknown, but must first be estimated judgmentally, unlike the MCS. Despite this difference we can still extract a stable A for the problem, thus demonstrating that the model can be estimated, even with minimal real-world data. In the first study, nobody was actually selected or rejected, but in the second they were. The second study thus provides an opportunity to present two metrics which describe the *level* of age discrimination. We call these the (i) discrimination index (I_D_), and (ii) wastage metric (W).

### Development in the classroom: A worked example

MCS is a longitudinal study following the lives of a sample of nearly 20,000 children born in the year 2000–2001, carefully stratified to represent the UK population [32–34]. We selected data from the “Forth Sweep” [*sic*] collected in 2008; specifically children born between 1 September, 2000 and 31 August, 2001 inclusive, but only in England and Wales because there is a clear boundary on which the school year starts, 1 September, which is not shared by Scotland and Northern Ireland. As part of the study, children were assessed by teachers on a range of academic qualities using Likert-type scales (1 = well below average, 5 = well above average). To illustrate the estimation of A, we will focus on their performance in terms of Speaking and Listening.

In the left-hand panel of Table 1 are the frequencies with which teachers classified students’ speaking and listening performance as *well below* [average], *below*, and so on. The right-hand panel contains the cumulative frequencies; for instance 267 September born children were rated above average or well above average (“≥4” given by 195 + 72). Note two points. First the column "≥ 1" is effectively all children born in the month (*e*.*g*., there were 470 September-born children surveyed). Second, because of stretched resources among teachers who were not central to this cohort study, this classroom data was only provided for a less than half of the children.

Next, the cumulative frequencies in Table 1 were expressed as probabilities (proportions) in Table 2. For instance, in the first row (Sep), for column "≥2", 0.9936 = 467 / 470. Treating these as upper tail probabilities, their corresponding z-scores are presented in the right-hand panel. For instance, p(z ≥ −2.4902) = 0.9936; mathematically: z = Φ^-1^(1–0.9936); or in Excel: normsinv(1–0.9936). Finally, the last column contains the t_B_ values for the mid-points of each month in [0,1]. For instance, for November it is (30 [Sep] + 31 [Oct] + 30/2 [mid-Nov]) / 365 = 0.2082.

**Table 2:** Transformation of cumulative frequencies from Table 1 into probabilities and z-scores.

	Upper tail probabilities				Z-Scores for Upper tail probabilities				Month Points
	≥ 2	≥ 3	≥ 4	≥ 5	≥ 2	≥ 3	≥ 4	≥ 5	
Sep	.9936	.9106	.5681	.1539	-2.4902	-1.3447	-.1715	1.0228	.0411
Oct	.9710	.8951	.5179	.1362	-1.8954	-1.2541	-.0448	1.0977	.1247
Nov	.9873	.9112	.4947	.1395	-2.2357	-1.3482	.0132	1.0824	.2082
Dec	.9808	.8699	.4307	.0938	-2.0708	-1.1261	.1746	1.3176	.2918
Jan	.9802	.8484	.4791	.1099	-2.0583	-1.0294	.0524	1.2271	.3767
Feb	.9696	.8454	.4169	.0843	-1.8743	-1.0170	.2099	1.3767	.4575
Mar	.9749	.8562	.4110	.0913	-1.9580	-1.0632	.2251	1.3326	.5384
Apr	.9713	.8541	.4091	.0742	-1.9001	-1.0540	.2299	1.4455	.6219
May	.9610	.8257	.3463	.0688	-1.7625	-.9373	.3952	1.4847	.7055
Jun	.9494	.8115	.3218	.0575	-1.6393	-.8834	.4626	1.5764	.7890
Jul	.9453	.7687	.3433	.0821	-1.6007	-.7344	.4035	1.3912	.8726
Aug	.9668	.8031	.2634	.0614	-1.8351	-.8526	.6328	1.5433	.9575

**Fig 3:**
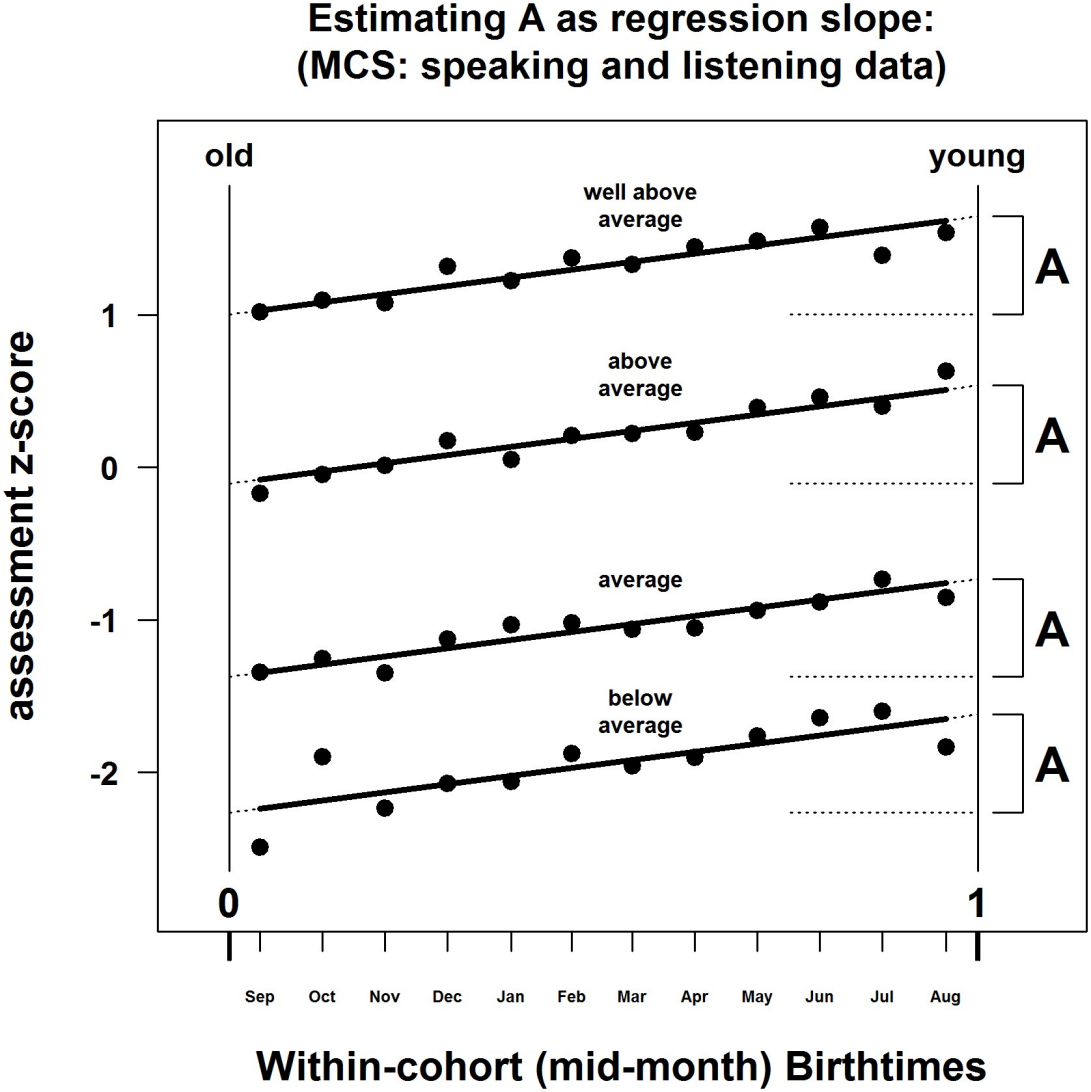
Estimating A as regression slope: MCS data for *speaking and listening*.

To recapitulate these ideas graphically, in Fig 3, the intercepts of the four lines at t_B_ = 0 can be interpreted as four values of C, namely four different criteria by which the oldest are to be judged as either *below average*, *average*, *above average*, or *well above average*. The slope of the lines can be interpreted as values of A. But how are A and C to be estimated?

One way would be to subtract the z-scores for August from the corresponding z-scores for September, much as was done in the illustrative example. This gives four estimates of how much progress has been made over those 11 months (beginning mid-Sep, ending mid-Aug). The estimates are: 0.6552, 0.4921, 0.8043, and 0.5204 for columns labelled ≥2, ≥3, ≥4, ≥5, respectively. Once averaged and scaled by 12/11 to cover the full year, we get A = 0.6742. Although simple to apply and understand, data for months October to July remain unused. A second approach is to regress the z-scores on the mid-month points for each severity of selection in turn. This gives slopes of: 0.6716, 0.5965, 0.7441 and 0.5583 for columns ≥2, ≥3, ≥4, ≥5, respectively, which average to A = 0.6426.

A more comprehensive, powerful but flexible approach is to run a single regression over all data. In it, the four columns of z-scores are stacked to provide 48 observations of the dependent variable. The independent variables are: mid-month t_B_, replicated four times for each attainment level; and a set of dummy variables to indicate at which attainment level the ratings had been made (≥3, ≥4, or ≥5, with ≥ 2 serving as the reference condition). Data are given in this regression-ready format for all MCS analyses reported here (see S1 Appendix), which also contains R code for the regressions. It gives A = 0.6428. Thus, for these particular data, all three methods triangulate well, particularly methods two and three.

#### Results for curriculum qualities

According to the RAE, as we move through the school year from September to August the proportion of students that the teacher has “good things” to say about should decrease, true at each attainment level; and this decrease would be indicated by a z-score that increases, thus leaving less probability mass to its right. Therefore, according to the RAE, we expect a *positive* sign on t_B_.

First, we examine the eight questions covering curriculum attainment. Table 3 contains the regression coefficients for birthtime t_B_, together with their associated t-values and p-values. Omitted from the table are the coefficients for the attainment-level dummy variables, which just tell us the self-evident, that fewer pupils reach higher levels of attainment. In Fig 3, the dummies shift the overall vertical location of the four lines marked *below average*, *average*, *above average*, and *well above average* relative to each other. For all eight curricular qualities, the linear term in t_B_ was significant. In Fig 3 the thick, continuous regression lines for each level of attainment have been extrapolated to meet the vertical dotted lines of t_B_ = 0, and t_B_ = 1. These are the estimated selection z-values for being born right at the start of the year (hence = C), versus end of the year, respectively. The difference between these z-values is therefore A, how many standard normal deviates the population of children of this age advance in a year. Alternatively, we can just read off the regression coefficient on t_B_ as our estimate of A.

**Table 3:** Regression coefficients for the eight curriculum subjects.

Subject	Model 1				Model 2	Model 3
	birthtime (= A)	t(43)	P	R^2^	F(1,42)	F(3,40)
Maths and number	0.774	17.01	<10^−19^	0.995	<1	1.80
Science	0.668	12.55	<10^−15^	0.995	<1	<1
Reading	0.657	17.93	<10^−20^	0.996	<1	<1
IT	0.650	9.36	<10^−11^	0.994	1.56	<1
Writing	0.643	14.43	<10^−17^	0.995	<1	<1
Speaking & listening	0.643	13.08	<10^−15^	0.995	2.32	<1
Expressive & creative	0.504	7.20	<10^−8^	0.993	<1	1.53
Physical Education (PE)	0.398	6.71	<10^−7^	0.995	<1	4.40*

* p < 10^−2^.

Model 1: z = b_0_ + b_1_t_B_ + Ʃ d_i_D_i_

Model 2: z = b_0_ + b_1_t_B_ + Ʃ d_i_D_i_ + b_2_t_B_^2^

Model 3: z = b_0_ + b_1_t_B_ + Ʃ d_i_D_i_ + Ʃ f_i_ (t_B_ * D_i_),

where d_i_ are the regression coefficients for the attainment-level dummy variables D_i_; and f_i_ are the regression coefficients for the birthtime x attainment-level interaction terms.

The single OLS approach yields additional useful information relevant to the assumptions behind TTG. When a quadratic term t_B_^2^ was added, there was no significant increase in model R^2^ in any of the eight subjects, meaning there are no non-linearities, and no seasonality at work. See column heading "Model 2" in Table 3, which are the F-tests for the change in R^2^ (ΔR^2^) associated with adding a quadratic term to Model 1. None was significant: p > .10 for all analyses. Second, a birthtime x assessment-level interaction was added, but again ΔR^2^ was non-significant for all qualities except PE, meaning that the slopes of the lines for each attainment level did not vary. See Fig 3, and the column heading "Model 3" in Table 3. Taken together, these results suggest Model 1 is preferred over Models 2 and 3. Qualities all advance at a constant rate (in z) over the year, whose rate (regression slope) does not vary between levels of aptitude. Therefore, it is legitimate to interpret the regression coefficients for t_B_ quite simply as the desired values of A, as we have above.

Finally, the proportion of pupils at each ability level is not strictly independent. For instance, if a greater proportion of September-borns are rated as “well above” average ability, by definition, a smaller proportion must have attained lesser levels. So, the regression analysis was repeated using (by-month) clustered standard errors instead. Results were highly consistent with OLS reported here.

Results reveal that A ranges from 0.774 (Maths and number) to 0.398 (Physical Education). So while mathematical skills advance at nearly 4/5 of a standard deviation across this cohort year, physical education advances much more slowly, at around 2/5 of a standard deviation. Interestingly, the traditional “3Rs”, as Reading, wRiting and aRithmetic are often known, appear to advance at similar rates, averaging out to A = 0.691. In practical terms, when sitting competitive examinations (which often rely heavily on ability in the 3Rs) for limited places at academically selective schools, account should be taken of a child's age because a year can make a notable difference. However, recalling that a small A indicates the lesser importance of between-age to within-age ability, if the same child performs poorly in PE, then maybe they just don’t have the necessary physical aptitude to be a star quarterback or make it onto the track team. They will improve, but only modestly with age.

#### Results for non-curriculum qualities

Teachers in the MCS were also asked about a series of non-curriculum related qualities. We focus on two that are not so explicitly taught, being more akin to personality traits than abilities. One is the development of empathy and pro-social behavior (see Table 4, items E1-E4). The other is the development of attention and focus, which are disaggregated into physical focus (F1-F2) and mental focus (F3-F5). Teachers answered these questions on a three-point scale: *not true / somewhat true / certainly true*. We reverse-coded F1-F3 for purposes of analysis, so as to preserve the sense of positive values of A being linked with positively valued qualities. We analysed the data following the same approach outlined above. Model 2 and Model 3 follow on from Model 1 to test, respectively, for non-linearities in the progress of qualities; and different slopes, or rates of advancement, at different levels of attainment. In all cases the simple linear Model 1 with constant slope is preferred (p > .10 for all tests of ΔR^2^ in Models 2 and 3).

**Table 4:** Regression coefficients for non-curriculum qualities.

Quality	Model 1				Model 2	Model 3
	birthtime (= A)	t(21)	P	R^2^	F(1,20)	F(1,20)
E1. Is considerate of other people's feelings	0.010	<1	>0.50	0.988	<1	<1
E2. Shares readily with other children	0.147	3.09	<0.01	0.990	1.96	<1
E3. Is helpful when someone is hurt, upset, or feeling ill	0.111	2.34	<0.05	0.991	2.08	1.27
E4. Often volunteers to help others (teachers or children)	0.127	2.69	<0.05	0.991	2.46	<1
F1. Is restless, overactive, cannot stay still for long (*R*)	0.195	3.58	<0.01	0.968	1.84	<1
F2. Constantly fidgets or squirms (*R*)	0.202	4.77	<0.001	0.977	<1	<1
F3. Is easily distracted, concentration wanders (*R*)	0.473	13.65	<10^−11^	0.991	<1	1.03
F4. Thinks things out before acting.	0.344	8.17	<10^−7^	0.995	1.17	<1
F5. Sees tasks through to the end, good attention span	0.573	12.28	<10^−10^	0.989	<1	<1

Note: (R) = reverse scored. Models as in Table 3.

Concentration, planning, follow-through (F3-F5) are all desirable qualities and we see that the ability of a typical child born at the very start versus the very end of the year advances by about half a standard deviation over the cohort-year (Ā = 0.463). The rate of advancement is slightly faster than PE, but definitely slower than the traditional 3Rs and related qualities. Indeed, mental focus might be seen as either a by-product or prerequisite in the acquisition of these traditional products of education. However, children who are restless and fidgety (F1, F2; Ā = 0.199) clearly learn very slowly not to be so. Interestingly, even less amenable to chronological development with age are the empathic qualities of E1-E4 (Ā = 0.099). Such development as there is must be barely noticeable to the observer, since empathic qualities advance at about a tenth the rate of height (mean A = 1.05, MCS dataset—not shown). This suggests that whereas you may *grow* taller, you simply *are* empathic (or not). Perhaps it is no wonder that psychotherapy, one goal of which is to develop a more empathic nature in the client, tends to be such a long process with often modest progress.

### English Premier League soccer academies

#### Estimating ability Advancement (A)

Typically, RAE has been researched in highly competitive sports such as soccer, where specialised training begins early for those who show promise, so it is befitting we should examine it here. This example differs from the last since we have substantially less data available for the TTG model, yet we can still infer A, the rate at which soccer talent advances annually. It is noteworthy that we are able to do this without ever understanding what exactly constitutes talent in this context, nor directly measuring the ability of any soccer players. In this example, children are not just rated but actually selected, so the concepts of age discrimination and talent wastage have real meaning. We will show how this important, yet practical, information can easily be derived from knowledge of the rate of annual advancement (A).

According to the Football Association (of England), during the competition year 2008–2009, which runs from 1 September to 31 August, 57%, 29%, and 14% of boys at English Premier League Academies were born in the first, middle, and last thirds ("terciles") of the year, respectively [35]. Similar percentages were found a football generation earlier [36], emphasising that the RAE bias is stable over time. We cannot work with these percentages directly, as we need to compute z-scores from percentages of the soccer-playing population, rather than percentages of the EPL academies. We estimated the number of academy boys to be 320 per annum (see S2 Appendix). So, in any EPL academy year-group we expect there to be 182.4 (= 320 x 0.57), 92.8 (= 320 x 0.29), and 44.8 (= 320 x 0.15) boys born in the first, middle, and last thirds (terciles) of the cohort year (T1, T2, T3). We also estimated the soccer playing population in England to be 100,000 in any year-group (see S2 Appendix). In the UK, births are nearly uniform across the year, so we could divide the 100,000 boys into three equal groups of 33,333. But since the birthrate drops slightly for T2 *and* there are fewer days in T2 (T1, T2, and T3 have 122, 120.25, and 123 days, respectively), we used per-day Office of National Statistics (UK) figures for 1995–2014 to make more accurate estimates of: 33,736 for T1 (Sep-Dec); 32,378 for T2 (Jan-Apr, weighting 29 Feb by 0.25); and 33,886 for T3 (May-Aug). Hence the selection probabilities for boys born in each tercile are estimated to be: 0.005410 (= 182.4 / 33736), 0.002866 (= 92.8 / 32378), and 0.001322 (= 44.8 / 33886), with corresponding z-scores of 2.548, 2.763, and 3.006, respectively (2.548 = Φ^-1^(1–0.005410), for instance). All the calculations are in S1 Appendix, where what-if analyses may be performed using alternative judgments.

**Fig 4:**
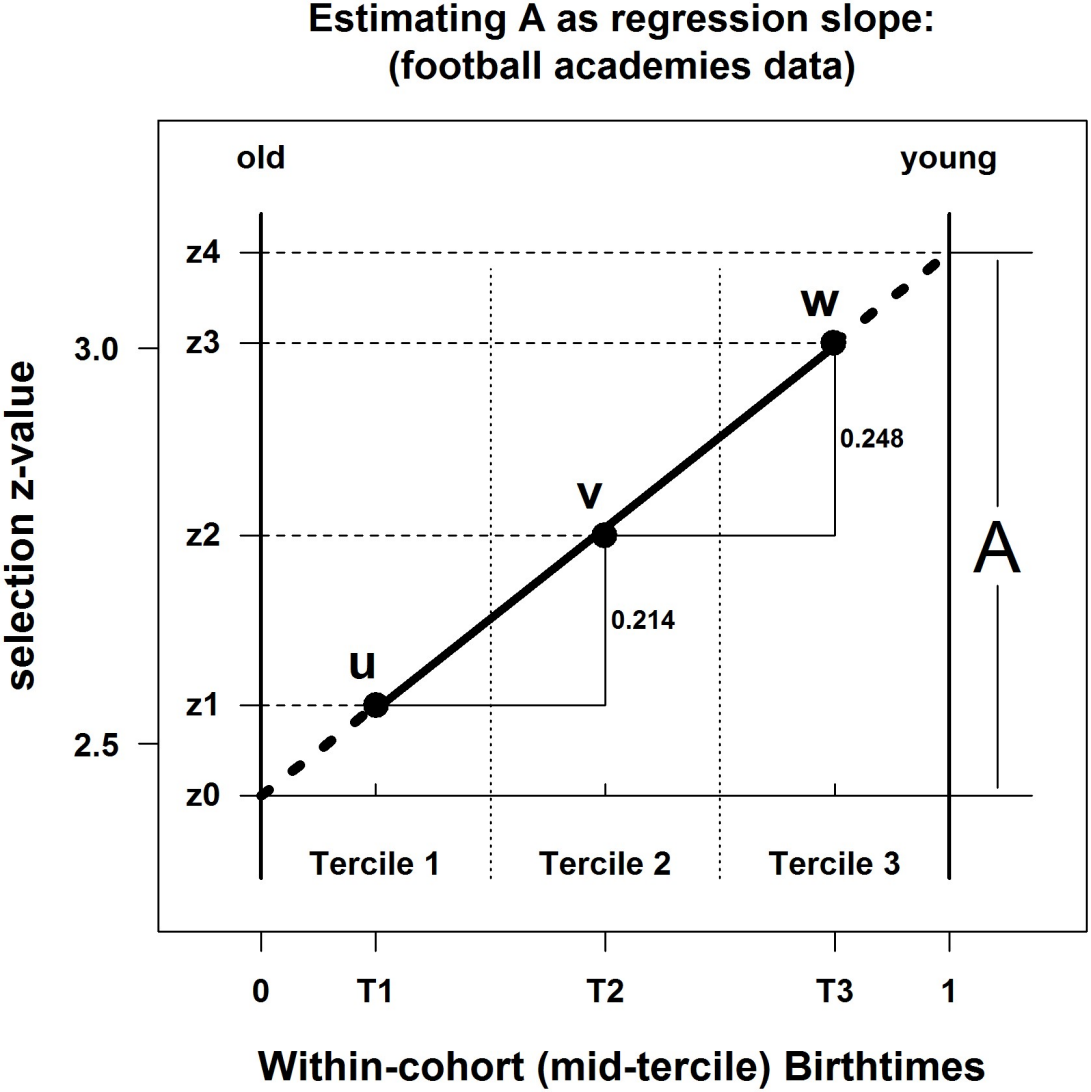
Estimating A as regression slope: Football academies data.

We make the usual assumption about grouped data, that the mean within an interval is its midpoint. Therefore, the three (t_B_, z) points, graphically presented in Fig 4, are then: (0.1670, 2.548), (0.4986, 2.763), and (0.8316, 3.006). From these we use OLS regression to estimate the equation:

$$z=2.429+0.689\;t_{B},$$

with R^2^ = 0.9987, and t(1) = 27.69, p < 0.05 for the test that the slope is non-zero. Hence A = 0.689 (slope), and C = 2.429 (intercept).

Because running regression with the bare minimum of 3 three observations is so unusual, an alternative approach would be to calculate the slope in Fig 4 between points u and v, and between v and w, then average; or to calculate the slope between u and w. In both cases A ≈ 0.689. Hence, the rate of annual advancement is effectively the same as that estimated via regression.

Naively, we might have expected football talent and physical education to advance at similar rates. However physical education in British schools tends to consist of a sundry group of sports and activities designed to give children exercise rather than to coach or teach to levels of excellence. The difference in A between professional football (A = 0.689) and physical education (A = 0.398; Table 3) may reflect the difference between incidental learning of general body-skills as children mature, compared with the focused, accelerated training involved in a highly valued, professional sport.

Since we are working with judgments about the effective population pool, it is prudent to perform a sensitivity analysis to understand how robust our calculations of A are to imprecision in the judgments we made. For instance, the number of academy players will not be exactly 320 per annum, though it will be close, but the figure of 100,000 aspirant players might have more imprecision attached to it. As a sensitivity analysis, we used 50,000 and 150,000 players as bounding estimates, yielding A = 0.740 and A = 0.663, respectively. In other words, though the starting population does make a difference, A is quite inelastic to changes in this assumption. In fact, a 1% increase in our estimate of the soccer population pool leads to less than a 0.1% change in our estimate of A.

The greater the annual advancement in ability (A), the greater advantage to older children, the greater the RAE, the greater the age discrimination, the greater the wasted talent, and so on. We can therefore use this estimated value of A descriptively to compare the severity of RAE between soccer nations, or to compare the severity of RAE *between* different qualities, as we did with the educational curriculum and non-curriculum qualities. Among the related statistics that can be derived from knowing A, we use it to calculate the selection probability of someone born right at the very start of the year versus someone born right at the end of the year, a kind of index of age discrimination. The midpoints of the first and last thirds of a year are each 2 months in from these extremes, meaning that the 4.07 ratio (57% versus 14%) quoted by the (English) Football Association [35] above, does not describe the full extent of RAE age discrimination as we show below.

#### Age discrimination

Given the regression equation z = 2.429 + 0.689 t_B_, estimated above, we use the values t_B_ = 0 and t_B_ = 1 to estimate the selection z-values for oldest possible and youngest possible in the cohort. They are 2.429, and 3.118. Thus, 0.7577% (= 1−Φ(2.429)) of oldest-possible boys will be selected, whereas only 0.0912% of youngest-possible boys will be selected. So, the extreme-oldest are about 8.31 times more likely to be selected by English Premier League soccer clubs than the extreme-youngest, which is twice as discriminatory as the 4.07 we computed by comparing terciles 1 and 3. We call this ratio the Index of (age) Discrimination, I_D_ = p(Oldest possible) / p(Youngest possible).

From the discussion above, we expect the estimated value of A to hold specifically for U9s. And so it should, because the pattern probably originates from that age. In fact, it may result from decisions made midway through the previous year, when the oldest boys were about eight and a half years old, the youngest seven and a half years old, and coaches made their selections for the up-coming year's cohort. These decisions, in turn, will have been informed by how the boys performed at the start of the season when they were between 7 and 8 years old. For this reason, we will assume that both the MCS and FA studies refer to the same age, implying it is valid to compare them.

#### Wastage

**Fig 5:**
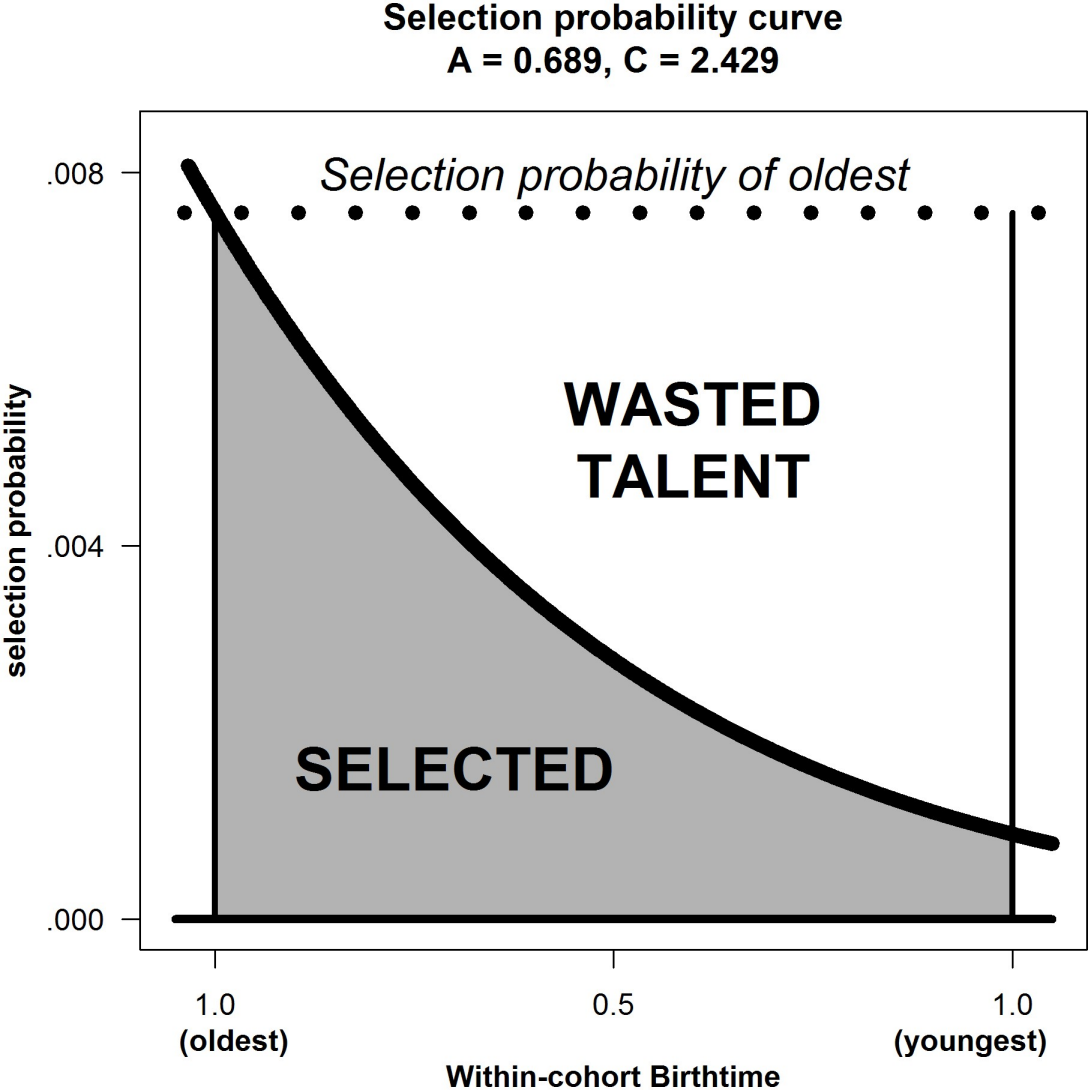
Index of age discrimination and talent wastage: Evidence from English Premier League Clubs.

While I_D_ focuses on the individual, a policy maker might be more concerned with how much talent is being wasted in the entire cohort because of RAE. We argue that the selection probabilities for the oldest should, in a non-RAE world, be uniform across the year (the dotted horizontal line in Fig 5). This would imply that regardless of whether children were born earlier or later in the cohort-year, they have the same chance of selection or attainment. Any shortfall from that is wastage. Connecting this back to Fig 1, each grey area to the right of the selection criterion now becomes a vertical line from the x-axis to the selection probability curve in Fig 5. Similarly, connecting this back to Index of Discrimination (I_D_), the line on the extreme left of the grey area (t_B_ = 0 for the oldest) is 8.31 times the length of the line on the extreme right (t_B_ = 1 for the youngest). Note that wastage at any particular age is measured by the distance from the horizontal dotted line to the selection curve, so that wastage for the entire cohort is the white area above the selection curve but below the dotted line. We therefore define wastage (W) as the ratio of the White area / (White + Grey area). Analytical solutions to determine the size of these areas can be achieved by integrating (calculus) the Gaussian, or via numerical approximation in a spreadsheet.

It is also revealing to calculate from W the implied population expansion factor (PopEx) that we would gain by eliminating RAE altogether. For example, if W = 0.6667, we have wasted, two-thirds of our talent pool, and have selected and retained only a third of the talent we might have had. If there were no wastage with the current population pool (W = 0), there would be a certain pool of talent. To achieve that same size pool of talent under the current level of wastage (W = 0.6667) the population would need to be tripled, meaning that the population expansion factor (PopEX) is 3. The general formula is:

PopEx=1 / Grey, (1)

if White + Grey area = 1 when standardised.

Also revealing is the related population contraction factor (PopCon), which describes how RAE has contracted the effective population pool. It is as follows:

PopCon = 1 / PopEx = Grey = 1 – W. (2)

Returning to the English Premier League example, we know that soccer talent advances by 0.689 standard deviations per annum (A). When determining the Index of Discrimination, we also deduced via a process of extrapolation that z = 2.429, so that children born on day 1of the competition year had a 0.758% chance (7.58 per 1000) of being selected. Following some calculus (not shown), the analytic solution revealed values for W = 0.570, or 57% wastage; PopEx = 2.33; and PopCon = 0.43 or 43%. Surely, every head of their country’s soccer association, every club’s head coach would want to avoid working with a talent pool that was only 43% of what it might have been? On a more positive note, who would not want to expand the effective pool of talent coming through their youth development system by 2.33 fold, simply by eliminating RAE?

## Discussion

This paper develops the TTG (Tails of the Travelling Gaussian) model to account for the origins and form of the Relative Age Effect (RAE). The principal output of TTG is a measure (A) of the degree of *age* discrimination potential in a context, which results from *when* the child was born in the cohort year. In virtually every context that has been researched, RAE discriminates against the younger child by decreasing the chances of achieving their life goals (*e*.*g*., being accepted at an Ivy League university, invited to play for the New York Symphony Orchestra, becoming a Nobel laureate, a CEO of a blue chip company, competing in the Olympics, playing soccer for Manchester United, or simply coming top of the class). RAE often operates through early selection to open or close doors onto advanced programs. But more insidiously, having spent a childhood being outperformed by their cohort peers, RAE may act to depress the young child’s expectations so that they aim lower in life and thereby achieve less than they might have [12, 24].

Like all forms of discrimination, RAE should be a salient issue for policy makers, managers, organizational recruiters, college admissions tutors, as well as scouts and coaches of sports teams, who are all entrusted to find and nurture talent. But until now, the literature reveals no viable measure to capture the extent of RAE operating in different contexts, thereby limiting our understanding of how pervasive RAE is; how aggressively it operates in different contexts; how it diminishes with children’s chronological age; and whether it has diminished in recent years.

The TTG model proposes that a particular *quality* (Q) a group of children possess at a specific chronological age follows a Gaussian (*i*.*e*., normal) distribution. Q may be a raw ability (*e*.*g*., running speed), a nurtured talent (*e*.*g*., playing a violin), or an attribute/characteristic (*e*.*g*., height). As the group gets older, their development on Q increases (they run faster, are more proficient at violin, get taller), so that their Gaussian travels along the Q axis. In any given cohort (typically a school year or competition year) there will be a Gaussian centred at Q_0_ for the youngest, one at Q_1_ for the oldest, and one at each point in between (Q_t_, with 0 ≤ t_B_ ≤ 1). By examining the proportion of children who exceed a certain criterion in each age group, we are able to infer the location of each group’s Gaussian along the Q axis. This allows us to determine how far the Gaussian travels over the year; or more simply how much they grow (in speed, in musical ability, in height). This distance is known as the rate of annual advancement (A), and because A is measured in standard deviations or z-values of the Gaussian, it is directly comparable with other values of A computed from widely different contexts, and in different data formats. It is also possible to examine whether growth has been accelerating, decelerating, or has it been linear (constant) over the year, as in all the examples studied here. One important point is that in TTG model, the value of A does not vary if the selection criterion is made more severe or more lax.

Analysing RAE data by TTG is a definite advance over past practice which has largely been descriptive with some simple statistical testing, but lacking any real attempt to develop a mathematical model of the underlying mechanisms in RAE. There are many advantages to TTG.

1. The plausible assumptions of a Gaussian distribution to quality and linear growth (travel) of the Gaussian with time / age act to demystify RAE so that its appearance and general form seem no longer anomalous, but almost inevitable. The assumptions are testable too.
2. We have shown that mean t_B_, ${\bar{t}}_{B}$, is anything but straightforward. An extreme ${\bar{t}}_{B}$ may be due to an extreme rate of annual advancement (A), or a severe selection criterion (C). ${\bar{t}}_{B}$ is not a measure of RAE: A is. Thus, research that seeks to explain severity of RAE by using ${\bar{t}}_{B}$ may be confounded. Similarly, ${\bar{t}}_{B}$ cannot be used as an independent variable and interpretation of its effect on the dependent in any simple way.
3. Knowing A, the rate of annual advancement, we can compare across disparate contexts, without need for further calculation. This enables the systematic development of knowledge in the whole field of RAE research, where previously it has remained fragmented. Additional value may be added by being able to compare values of A from different contexts, as we have done briefly in reflecting on learning in the 3Rs (Reading, wRiting, aRithmetic) versus learning to empathise; or in reflecting on the developments of elite football talent and physical education (PE) as an ensemble.
4. Once A and C are known, derivative statistics may be calculated, such as I_D_, the index of (age) discrimination, and W (the proportion of wasted talent), as well as the closely related PopEx and PopCon. We suggest that it is these kinds of metrics that will be of most value to practitioners who are often interested in ascertaining the scale of any gap between most advantaged and disadvantaged in a cohort. One can draw parallels between I_D_ and other metrics such as the Gini coefficient, widely used to compare income inequality or market share concentration in different countries.
5. Because TTG is a formal model, it supports what-if scenarios that decision makers (school principals, sports academy managers, orchestra conductors, even government ministers) can use to understand the consequences of policy changes. Being able to generate managerially relevant and insightful statistics helps here too. Being able to simulate, the TTG also provides the mechanism to generate testable hypotheses that other researchers may examine.
6. It is worth reflecting on how we might have designed a field study to estimate A for football talent. First, what are the dimensions along which talent exists, and how should they be combined? Then there is the problem of gaining access, and the considerable leg-work involved in collecting, collating and analysing the data. By contrast, all TTG needed was five numbers: three publicly available percentages and some background knowledge to judgmentally estimate the two population parameters. Of course, the real field-work was done by the dozens of coaches whose implicit models of talent, which they would "know when they saw it", summed collectively to yield the percentages; or by the hundreds of teachers who assessed their pupils’ academic abilities in the Millennium Cohort Study.
7. TTG is very flexible given it applies whether the quality just happens to someone, such as height or skin tone, or whether it is a lot more effortful, such as learning to read; whether it is easy to measure (such as running speed, maths), or not (such as acting talent); or whether it is small (empathic response) or large (again, maths). Also, TTG's inputs can be detailed information (as in our academic example), or very scant information (as in our football talent example). The levels of measurement of the data may be ratio (height), ordinal (teachers rating their pupils on a Likert scale), or even simple counts and percentages (football academies). Yet each produces the same key statistic, the rate of annual advancement (A), that is directly comparable.
8. When only the high-achieving right tail of the Gaussian is known, its distribution may need adjusting to control for known non-uniformity in the population birthrate across the year [37]. TTG can easily adjust its extreme-tail distribution relative to the baseline population if this differs from uniform, exactly as we did in Study 2. A similar adjustment could also be made if ever seasonality in a subpopulation were shown to be associated with RAE, therefore opening the door to a counter-explanation about RAE's origins [38]. In S3 Appendix we argue that subpopulation seasonality in birthrate is unlikely to have been operating in either of these datasets, or to be able to re-explain the current RAE literature.

None of these advantages is present in previous models of RAE or methods of RAE research. We therefore believe that the TTG maybe a spur to properly understand, quantify, integrate past and future research in this important area, as well as to devise solutions for its mitigation, to the advantage of not only those who suffer RAE age discrimination, but also for those who wish to maximise talent in whatever field.

One reason that makes RAE so noteworthy is finding that it is present in places where it really shouldn't be present; for instance, among elite sport-persons, and entrants to Oxford and Cambridge Universities. Should the advantage of being oldest in the class not have worn off by then? In the former case, RAE may exist because of early selection into talent schools, which never get corrected as the *intrinsic* advantages of being older wear off. So one interest in focussing on 7 to 8 year olds is that selection into advanced talent schools often takes place this early on, resulting in a massive bias towards accepting older children; the bias tends to be self-sustaining thereafter, as RAE research attests. In the latter case, RAE is sustained by older children doing better in exams, thus going to better schools, being in more able work groups, and by the virtuous (or vicious) cycles of self-esteem engendered by parents, teachers, peers, and the child themselves, all based on RAE underperformance [24]. A formal model of RAE such as the TTG can be extended to take a long-run view of how these phenomena work.

Finally, a serviceable model can sometimes be used in situations that it was not originally intended. In this respect, the TTG model may contribute to scientific understanding in gerontology, specifically the deterioration of faculties in older age. Here, the rate of annual “advancement” would in fact be decline, and A would take on negative signs. But just as a set of As supports a discourse on the various qualities that make up child development, so too could A provides the tools for another discourse on the loss of different abilities as we age. Fortunately, it seems that the TTG model is still young and dynamic, with plenty of life ahead of it yet.

## Supporting information

S1 Appendix Regression data, R code, and sample R output. (XLS)

S2 Appendix Proportion of boys in English Premier League academies. (DOCX)

S3 Appendix RAE and seasonal fluctuations in subpopulation birthrates. (DOCX)
