# Supplementary material for: Tails of the Travelling Gaussian model and the relative age effect: Tales of age discrimination and wasted talent
Source: PLoS One. 2017 Apr 20;12(4):e0176206. doi: 10.1371/journal.pone.0176206 (PMC5398632; doi:10.1371/journal.pone.0176206)
Supplement: S2 Appendix — (DOCX) [file pone.0176206.s002.docx]

**Appendix 2: Proportion of Boys in EPL Academies.**

First, we need to estimate the proportion of boys selected into academies. It is the number selected divided by the number eligible. Each term in this ratio is estimated judgmentally.

*How many boys per annum join an academy?* If we estimate that each of the 20 Premiership football clubs runs a squad of 16 outfield players (to fill two 7-a-side teams with two subs per team), then we arrive at a grand total of 320 boys per annum who enter Premier League academies.

*How many boys per annum are eligible for selection?* Office of National Statistics (ONS) figures suggest nearly 400,000 boys are born in England per annum. Naively, we might think that the selection percentage is therefore 1k / 400k = 0.25%. However, not all boys play football; or if they do, they or their parents may not want them to play academy football, thus making them ineligible. Guided by percentages given in a recent report by UK's Department of Education [33], that about half of children aged 8 or 9 participate in at least 3 hours of out-of-school sport, coupled with the assumption that only half of those will be playing football with any seriousness, we make another ball-park estimate of 100,000 active football players in the UK at the relevant academy-selection age, again rounded to emphasise it is an approximation.

To triangulate this estimate, we turn to Wikipedia's entry *Football in England*, (<http://en.wikipedia.org/wiki/Football_in_England#Youth_leagues>). They claim there are 4,000 clubs that participate in the FA Youth Cup for U18s, organised by the FA. At an average of 15 players per squad, we arrive at an estimate of 60,000 serious players at that kind of age. Extrapolating backwards, this figure is likely to be a lower bound if we used it to estimate the number of boys playing at U9s, because of the steady drop-out that occurs through the teenage years. Furthermore, Wikipedia claims there are "over 40,000 association football clubs." If 4,000 of these appear in the FA Youth Cup, then 36,000 do not, and among their number must be many that run just junior teams. In conclusion, the real figure for eligible U9 boys playing football should definitely exceed the 60,000 estimate.

In summary, we estimate that there are 320 academy players and 100,000 eligible players, making a selection criterion of 0.32% for entry into an academy, approximately 1 in every 300. But of course, being an average across the year, the proportion selected will be more generous to older boys in the cohort, and less generous to younger ones. Given the uncertainty involved in estimating academy numbers, and eligibility numbers, we propose to also analyse using pools of 50,000 and 150,000 eligible players, as well as 100,000.
