# Supplementary material for: Tails of the Travelling Gaussian model and the relative age effect: Tales of age discrimination and wasted talent
Source: PLoS One. 2017 Apr 20;12(4):e0176206. doi: 10.1371/journal.pone.0176206 (PMC5398632; doi:10.1371/journal.pone.0176206)
Supplement: S3 Appendix — (DOCX) [file pone.0176206.s003.docx]

**S3. Appendix**

**RAE: A seasonal effects in subpopulation birthrates, or an organizational artefact?**

Seasonal fluctuations in the population birthrate can easily controlled for in TTG, as we have in Study 2, to reveal RAE uncontaminated by seasonality. A more subtle possibility is that birthrate may fluctuate differently for different socio-economic groups that each nurture different kinds of children with different interests and propensity to different activities [a]. Then, at least in theory, RAE could be driven by genuine seasonality in subpopulations. To put the argument bluntly, suppose there is a football playing class whose births peak at the start of football's competition year, whether by design or otherwise. If so, football-related RAE might be driven by this subpopulation, whose instrumental rôle could easily be masked in the overall population, and similarly for other activities. In this way, both Study 2 *and* Study 1 would be vulnerable to reinterpretation.

For this argument to have any force, sizeable seasonal differences must first exist in subpopulations. But in the UK at least, such differences have not been found, either historically [b], nor in recent data [c]. Moreover, in US data, where subpopulation seasonalities have been observed [a], fluctuations tend to be small percentage-point differences, rather than the order of magnitude differences typically found in RAE. This disparity casts doubt on subpopulation seasonality being the sole drivers of RAE.

Nonetheless, it could still be argued that the critical subpopulations have yet to be identified. Perhaps they are more related to personality or physiological differences than demographic ones? One problem, even for such an ill-formed and therefore highly malleable argument, is that school years start at different times in different countries and different states of the US [d]. These provide a natural experiment with counterbalanced design, yet RAE is ubiquitous across all school starting dates. Also, as mentioned earlier, RAE and genuine seasonality have different signatures we expect of their frequency distributions across the year (discontinuous sawtooth versus continuous wave). The RAE literature provides copious examples that conform to a sawtooth, but almost nothing that conforms to a wave-like distribution. But even here it could be argued that it is by the design of family planning that subpopulation fluctuations come about, as parents proactively seek to optimise their child's future in directions that they (the parents) value. Family planning consequences could be discontinuous across year start-dates.

Perhaps the clinching argument, however, is that when the starting date of the competition year is changed (another natural experiment), RAE almost immediately snaps into a pattern consistent with the new year rather than the old one: [e] for Australian data; and [f] for Belgian data. This rules out family planning as an explanation, because several years previously parents would have planned for the wrong (old) competition year; yet it is the new one that shapes the form of RAE. In conclusion, while genuine seasonality may yet play some small part in RAE, either to mask or enhance it, the dominant factor is in the arbitrary definition of the school or competition year. In short, RAE is still an organizational artifact.

Subpopulation fluctuation in birthrate is an issue to be raised about RAE in general, and not specifically with TTG. But if it were ever substantiated that subpopulation fluctuations might be important in RAE, then TTG could take this into account by changing the baseline seasonality of births against which RAE is measured. In Study 2 we adjusted that baseline for terciles from the naive (1/3, 1/3, 1/3) to (0.337357, 0.323784, 0.338859), which accounted for population seasonality in the different tercile. Anlogous calculations could adjust for subpopulation seasonality.

References for S4.

[a] Buckles KS, Hungerman DM. Season of birth and later outcomes: Old questions, new answers. Review of Economics and Statistics. 2013; 95(3): 711-724.

[b] James WH. Social class and season of birth. Journal of Biosocial Science. 1971; 3: 308-320.

[c] Goodman R, Gledhill J, Ford T. Child psychiatric disorder and relative age within school year: cross sectional survey of large population sample. BMJ, British Medical Journal (Clinical Research Ed.). 2003 Aug 30; 327(7413): 472 - 475.

[d] Bedard K, Dhuey E. The persistence of early childhood maturity: International evidence of long-run age effects. The Quarterly Journal of Economics. 2006; 121(4): 1437-1472.

[e] Musch J, Hay R The relative age effect in soccer; Cross-cultural evidence for a systematic discrimination against children born late in the competition year. Sociology of Sport Journal. 1999; 16: 54-64.

[f] Helsen WF, Starkes JL, van Winckel J. Effect of a change in selection year on success in male soccer players. American Journal of Human Biology. 100; 12: 729-735.
